# Engaging Without Exposing: Use of a Fictional Character to Facilitate Mental Health Talk in Focus Groups With Men Who Have Been Subject to the Criminal Justice System

**DOI:** 10.1177/1049732318785359

**Published:** 2018-08-01

**Authors:** Christabel Owens, Mary Carter, Deborah Shenton, Richard Byng, Cath Quinn

**Affiliations:** 1 University of Exeter, Exeter, United Kingdom; 2 Plymouth University, Plymouth, United Kingdom

**Keywords:** mental health and illness, marginalized or vulnerable populations, prisons, prisoners, qualitative, focus groups, vignette, Britain

## Abstract

In an effort to encourage men with experience of being subject to the criminal justice system to contribute to focus group discussions on the sensitive topic of mental health, while also doing our utmost to protect them from discomfort or risk of exploitation, we used a novel technique involving the creation of a fictional character, supplemented by an audio-recorded vignette. We studied the role played by this technique in achieving our stated aims of “engaging without exposing.” In this article, we report on the use of this technique in three focus groups, showing how in very different ways it shaped the interaction between participants and generated crucial insights into the lives and service needs of each group. We conclude that the technique may lend itself to being used in focus groups with other marginalized or seldom-heard populations.

## Introduction

People who have been subject to the criminal justice system can be regarded as a seldom-heard group, whose opinions are rarely sought (Jayne, 2006). The term "seldom-heard" is increasingly being used to denote groups of people who are typically left out of research and denied opportunities to participate in service development activity (Morrow, Boaz, Brearley, & Ross, 2012; Robson, Sampson, Dime, Hernandez, & Litherland, 2008; Ryan, Hislop, & Ziebland, 2017). This term is now preferred to the more traditional “hard-to-reach,” which can imply that members of particular groups are being deliberately evasive, and “vulnerable,” which is ill-defined and contestable, as those who are deemed by professionals to be vulnerable may not see themselves as such (Aldridge, 2014). The term “seldom-heard” lacks any implicit value judgment and places the responsibility for *hearing* the voices of currently excluded groups squarely on the shoulders of researchers and service providers. Those who accept that responsibility may need to adapt their methods to suit the particular needs of those groups and ensure their safety.

In terms of health and social care provision, those with experience of being subject to the criminal justice system^1^ have been described as one of the most disadvantaged groups (Jayne, 2006). Very high rates of mental health problems have been recorded in this population, particularly in those in prison or recently released from prison (Brooker, Repper, Beverley, Ferriter, & Brewer, 2002), including high levels of suicide and self-harming behavior (Pratt, Piper, Appleby, Webb, & Shaw, 2006), post-traumatic stress disorder, personality disorders, substance misuse problems, and associated co-morbidities (Byng et al., 2012). Those leaving prison also experience a wide range of personal and social problems, including homelessness, unemployment, and loss of relationships with partners and children. There are complex associations between mental health, substance misuse, social exclusion, and criminal behavior, but these tend to be studied separately and interventions to address them are frequently developed and delivered in isolation. Moreover, while prison health care services have recently improved, access to and continuity of care on release from prison is extremely poor. Community services are often unaware of the needs of prison leavers and ill-equipped to meet them (Byng et al., 2012).

The aim of the ENGAGER project was to develop and evaluate a complex collaborative care intervention for men with common mental health problems, such as depression and anxiety, which would bridge their transition from prison to the community.^2^ The intervention was specifically designed to break down service barriers, particularly between health and criminal justice sectors, and build constructive relationships between professionals and clients, thus providing a foundation for ongoing engagement with services (Kirkpatrick et al., 2018). The intervention development drew on a number of sources, including a realist review of the scientific and gray literatures (Pearson et al., 2015), a series of detailed case studies of current practice, the lived experience of a group of peer researchers, and a formative process evaluation embedded in a pilot trial. However, to optimize the intervention, we identified a need for further knowledge relating to particular subgroups and issues. We therefore decided to convene a series of focus groups with purposefully sampled members of the target population.

Focus groups are rarely used with forensic populations, for reasons that have not been explicated but may have to do with perceived risks to either participants or researchers. We were keen to use focus groups rather than individual interviews because we were interested in shared beliefs as much as individual experiences. Focus groups offer the opportunity to obtain a group perspective on the topic in question and for new insights to be created through interaction between participants (Onwuegbuzie, Dickinson, Leech, & Zoran, 2009), notwithstanding the fact that participant interaction is rarely reported (Belzile & Öberg, 2012; Kitzinger, 1994) and the danger of seeing consensus where it may not necessarily exist (Barbour, 2007). Ryan and colleagues used focus groups as a means of finding out what mattered to people in other seldom-heard groups (Ryan et al., 2017), and viewed the method as relatively unproblematic. By contrast, we anticipated that there might be some challenges and risks in undertaking focus groups on the sensitive topic of mental health with men who have been subject to the criminal justice system. Broadly speaking, the challenges were twofold. The first was that of persuading men from the target population to participate in a discussion of mental health issues at all (*engaging*). In addition to the difficulties that men in general have with “troubles telling” (Jefferson, 1981; Johnson, Oliffe, Kelly, Galdas, & Ogrodniczuk, 2012), those who have been involved with the criminal justice system do not easily recognize their problems as being mental health related (Howerton et al., 2007). The second challenge was the opposite, namely that they might inadvertently put themselves at risk by disclosing personal information that could be exploited by others at a later date (*exposing*). The risk of over-disclosure in focus groups has been highlighted by others (Peek & Fothergill, 2009; Tolich, 2009), but was especially acute in our target population, among whom personal information functions as currency and can be traded. This risk increases when participants are recruited within a relatively small geographical area and may belong to related social groups. We were anxious to avoid putting anyone in a position where information they shared for the benefit of research could be used against them. The risk of self-exposure also applied to members of the research team. While the sharing by qualitative researchers of their own stories can help to build rapport with participants and create a “level playing field” (Dickson-Swift, James, Kippen, & Liamputtong, 2007), attention to personal safety is always important.

We saw it as central to our duty of care to engage participants fully, without exposing them or the researchers unduly. We sought to achieve this through a novel technique in which participants created a fictional character for use as a focus for discussion. In this article, we report on the use of this technique, with the aim of extending the literature on the safe and effective conduct of focus groups with seldom-heard groups, including those who have been subject to the criminal justice system.

## Method

### Recruitment and Composition of the Groups

We convened three focus groups, each designed to elicit the views of a specific subgroup of the overall target population and fill a particular knowledge gap in relation to intervention development. For each group, participants were recruited through a community-based service. Staff in these organizations played a crucial role in identifying eligible participants, approaching them on our behalf, encouraging them to turn up on the day, being available to discuss participants’ concerns beforehand, and supporting them afterward should they be distressed by the issues raised in the group. Recruiting through these organizations may have meant that the *most* seldom-heard, namely those who are not in contact with any statutory or voluntary service, remained excluded, but we believed that this was a necessary price to pay to ensure the safe and ethical conduct of the research. In each case, the organization through which we recruited allowed us to hold the focus group on their premises. We provided hot drinks, sandwiches, and biscuits; reimbursed travel expenses, and planned to give all participants a £20 high-street shopping voucher as an expression of thanks for their time and contribution, although in one group (Group 2 below) the terms of their sentence did not permit this. The study received NHS ethical approval from the National Research Ethics Service East of England Committee (13/EE/0249) and from the National Offender Management Service (2013-187). All participants gave informed consent to take part.

#### Group 1: Focus on homelessness

In Group 1, the focus was on the experience of homelessness following release from prison. Six participants, aged 25 to 60, were recruited through a local charity that provides vocational training and other opportunities for people recovering from drug and alcohol abuse, homelessness, offending behaviors, or mental ill health. All six participants had served prison sentences and had experience of homelessness, although none was homeless at the time of the focus group. Two of the participants were peer workers at the charity and were known in this capacity to some of the other participants; the remaining four did not know each other.

#### Group 2: Focus on youth

In Group 2, the focus was on the experience of younger men, aged 18 to 24, as they are significantly overrepresented in the criminal justice system (Britton, 2012) but were underrepresented in our various data sets. This subgroup was the hardest to recruit. We made prolonged efforts to do so through a probation service and eventually held the focus group with only two participants, although four had agreed to take part. Both participants had experience of being in prison, but at the time of the focus group were serving community sentences under probation service supervision. They did not know each other.

#### Group 3: Focus on immediate post-release period

In Group 3, the focus was on men’s experience of the period immediately following release from prison. Four participants were recruited through a drug rehabilitation project, all of whom had been released within the last month. Three men were present for the entire session; the fourth arrived half-way through. None of the men knew each other.

Participants of all three groups identified as White British, which was unsurprising as the groups took place in a part of the country with an underrepresentation of other ethnic groups.

### The Fictional Character Technique

Vignettes are often used within a focus group setting as a prompt for discussion and can be particularly helpful when addressing sensitive or contested topics, by focusing on a concrete example rather than an abstract concept, by avoiding questioning participants directly about personal matters and by allowing them to distance themselves from the topic as much or as little as they wish (Gott, Seymour, Bellamy, Clark, & Ahmedzai, 2004; Hodgins, Millar, & Barry, 2006; Holley & Gillard, 2018). The traditional vignette involves a written text depicting a person or scenario, which is either given to participants to read or is read aloud to them at the start of the session. We modified this technique to include a two-stage process.

First, the group worked together to create their own fictional character. They were given a set of eight pictures of men of different ages and ethnicities (taken from magazines and photocopied in color), and were asked as a group to choose one picture. This was pinned up on a flip chart. They were then encouraged to give him a name and to create an identity for him, deciding collectively on his age, marital status, background (including possible criminal history), and interests, and fleshing out his character as much as they wished. The details they provided were recorded alongside his photo on the flip chart, which was positioned so as to make it feel as though the fictional character was in the room with them.

Once this was complete, the group was asked to listen to an audio-recording and to imagine that it was the fictional character talking. In the recording, the character revealed that he was experiencing depression. The script (see the appendix) was initially drafted by two researchers (Quinn and Shenton) based on their extensive experience of interviewing men with experience of the criminal justice system about mental health issues. This was then shared with a young man who was known to the research team, who had been in trouble with the police, had learning difficulties, and had experienced depression. He was invited to suggest changes to the text that might make it more comprehensible to his peers. Speaking in a local accent, the young man then recorded the passage into a digital audio-recorder. After the participants had listened to the recording, they were asked to reflect on whether their perception of the fictional character and their feelings toward him had changed and if so how; what they thought he should do; how they would try and help him if he were one of their own mates; what sort of services there were in the local area for someone like him; and how accessible and adequate they were.

We intended that this technique would achieve several purposes. First, it was designed to act as an ice-breaker at the start of the session, allowing confidence to grow and enabling the group to become comfortable with one another by working on a shared activity, prior to discussion. It then allowed us to introduce mental health talk early in the session without requiring the participants to come straight out with disclosures about themselves and their own inner lives. By creating a fictional other and focusing attention on his problems from a third-person point of view, we intended to take the spotlight off the participants and thereby reduce feelings of exposure and consequent discomfort. We also hoped to draw out prejudices, stereotypes, and other ideas about mental health held by participants, either individually or collectively, that might not emerge if they only talked about themselves. Finally, we hoped it would help them to think beyond themselves and reflect more generally on what other people might find helpful.

### Conduct of the Groups

Each group was conducted by a team of three, consisting of a facilitator, a co-facilitator, and a peer researcher. The role and style of the facilitator has been described as pivotal in focus groups (Macnaghten & Myers, 2011), having the potential to create an atmosphere of “relaxed informality” in which participants feel comfortable to contribute (Puchta & Potter, 2004), while also requiring the ability to keep the discussion on track, probe deeply for the reasoning behind participants’ responses, and ensure that the full range and diversity of views is heard (Cohen & Garrett, 1999). Several authors have drawn attention to the need for at least one additional team member to be present, if only to note down any significant nonverbal behaviors and any issues to bring up in debriefing (Kidd & Parshall, 2000). We used the same facilitator (Quinn) and co-facilitator (Shenton) for all three groups to ensure consistency. Both were chosen for their ability to engage with the target population.

Peer researchers are defined as people with relevant lived experience, or members of the target population, who are invited to become part of the research team and play an active role in the design and conduct of a study. They can help to bridge the gulf between researchers and participants. By “speaking the same language” as participants they can help to open up communication, reduce power imbalance, and contribute an insider perspective to the interpretation of findings (Burns & Schubotz, 2009; Lushey & Munro, 2015). Men with lived experience of the criminal justice system had played a central role throughout the wider ENGAGER project, continually challenging the thinking behind the intervention and critiquing the trial science. For the focus groups, we considered it essential for a peer researcher to be present, particularly as the facilitator’s and co-facilitator’s female gender accentuated their social difference from the participants. Two different peer researchers, one for Group 1 and one for Group 3, were drawn from the ENGAGER Peer Researcher Group but, due to timing issues, no peer researcher was available for Group 2. Instead, a male member of the wider project team stepped in as proxy, to preserve the gender balance within the team and to perform the peer researcher tasks.

The peer researchers’ job was to get the group started by conducting the fictional character-building exercise, follow up the audio-recording with broadly predefined questions, and probe if a participant said something during the remainder of the discussion that the peer researcher judged to be either unclear or of particular significance. Each peer researcher was fully briefed beforehand about the aims of the focus group and his role in it, and was encouraged to think carefully about how much he should disclose about himself. He was also given an opportunity to debrief with the facilitator immediately after the group. During the focus groups, the facilitator used a more structured topic guide than she is accustomed to using, to give the peer researcher a sense of security and enable him to come in on cue at key points. A further two peer researchers helped with analysis and interpretation of data.

### Data Analysis

Focus groups were audio-recorded and transcribed verbatim. In each case, once the transcript was available, the facilitator met with a member of the Peer Researcher Group to work through it. The peer researcher clarified what was meant by particular terms and was able to shed light on both the content of the discussion and the nature of the interaction between participants. The content of the discussions was then analyzed rapidly to inform intervention development. For the purposes of this article, the transcripts were subsequently re-analyzed with a focus on behavior (verbal and nonverbal), to assess how well the fictional character technique had worked. This secondary analysis was carried out by two researchers (Owens and Carter) who had played no part in the running of the groups. They both independently listened to the audio-recordings while reading and re-reading the transcripts, and then coded the latter in terms of who was speaking (participant, facilitator, peer/male researcher), about whom (self, fictional character, other known person, others in general), how they were interacting (e.g., inviting agreement, concurring, challenging, joking), and how they were positioning themselves emotionally (e.g., expressing empathy, ridicule). Preliminary findings and interpretations were shared with the facilitator and co-facilitator, who contributed contextual details from their field notes, as well as insights gleaned from the peer researchers.

## Results

There were big differences between groups in respect of how the fictional character technique worked.

### Group 1

Group 1 had no hesitation whatsoever and immediately threw themselves into the task, taking little time to reach agreement on a picture. They selected a young White male, whom they named Simon, and volunteered plenty of descriptive detail about his shady past. They clearly identified him as someone with experience of the criminal justice system (in other words, “one of them”), drawing on their shared knowledge of criminal activity to flesh out his history. Simon was depicted as a prolific offender, who had served repeated prison sentences:

C1^3^: Two or three little ones and then a whopper.

A1: I’d say he’s got violence on his record. And a bit of fraud.

C1: Drugs, violence, driving convictions, that sort of thing.

E1: Sort of like petty crime, you know that sort of, what do they call it? Aw, what is it?

D1: Opportunist.

E1: Yeah, opportunist.

B1: Ooh, big words.

D1: Shall we just say low-level, low-level crime or something like that?

B1: No-one’ll know what that means.

A1: Petty crime, petty criminal. Preferential treatment, with two fs.

[General laughter]

This exercise continued for around 12 minutes, with the group obviously enjoying what they took to be a game. Some way into it, it emerged that they believed (mistakenly) that the pictures were of people who were known to the research team and that the identity they were assigning him might turn out to be wildly incorrect:

A1: Bit of a hard man, Jack the Lad, or whatever you want to call it. I bet he’ll be a doctor now, you wait.

C1: It’s going to be your office manager or something.

A1: Probably ends up going to church every Sunday.

There was much laughter, friendly banter, and joking, especially about Simon’s crooked nose, which they attributed to his love of boxing. What was interesting in this group, however, was that, even as they were enjoying depicting Simon as an “iron man” and a thug, they recognized that beneath the hard exterior there might lie deep insecurities and a vulnerable core:

P-R: What do you think his background might be like?

A1: He might have had a bad childhood

B1: Been in secure units, in care

E1: Poor education

P-R: Do you think he looks insecure, or something like that?

D1: He looks worried, din he. Really worried, like.

P-R: Worried, like stress?

D1: Yeah, stress, definitely.

This insight was confirmed for them as they listened to the audio-recording. Even as the recorder was being switched off and before they had been asked any questions, Group 1 participants voiced genuine concern for Simon:

C1: He’s depressed.

E1: There’s some serious mental health issues going on there.

There was an immediate shift in mood from the jocularity of the character-building game to one of gravity and sincerity. This was accompanied by an almost immediate switch from the third-person to the first-person point of view (from “he” to “I”), as the recording triggered a spontaneous disclosure by one participant that he had been in the same situation. His rich, figurative language reveals the depth of his identification with the fictional character:

A1: I think like, before I came here to be honest, I was in that situation. Sitting in a flat with the curtains drawn every day. [Other participants concur: Yeah, Mmm] Depressed, drinking until I’d fall asleep or black out or whatever. And I can see that he’s probably, without him saying it, that he’s probably lost his relationship, which is doing his head in. He’s a danger to himself because he’s probably addicted to something as well. Because he mentioned on there, wasn’t an alcohol thing but maybe could have been heroin or whizz or something. And like, he’s given up his self-esteem and his self-worth has gone right down the pan. And the only way is up, ’cause at the minute he’s as low as a snake’s belly, or under it even. That’s what I’d say.

This led into an extended discussion among the participants about the difficulties men experience in showing their feelings and their habit of “putting on a front,” with many examples being given of the ways in which they mask their distress:

F1: Me, I’m like I’ve got to put a front on . . . I can’t let people see I’m weak and crumbling, so I’ll go and sell my drugs. It’s putting the front on. Put the front on, put the front on . . . And it’s no different when you do go to prison. I still put the front on when I’m in prison. I ain’t going to let people see me weak in jail.

After a break, the group was re-started with a reminder of Simon’s situation and a series of prompts about what issues he might face when coming out of prison, particularly if he had no accommodation to go to. Again, the discussion flowed freely, moving seamlessly from third-person to first-person talk and back again. By constructing their fictional character in their own image, as “one of us,” the participants were able to alternate between “he” and “I,” depending on which felt more appropriate or safer for them at each point.

This group of men seemed comfortable with the group setting. They were ready to talk openly with each other and to engage in mental health talk, and it is possible that they would have been able to do so without the fictional character technique. Nonetheless, the fact that Simon remained “present” throughout the discussion suggests that he played a useful role. He provided an anchor point that was used by the research team in two main ways, first, simply as a convenient peg on which to hang questions. Using the character in this way avoided asking participants directly about themselves, which might have caused them discomfort. It allowed them instead to project their feelings onto a hypothetical other. For example,

P-R: What type of effect is it gonna have on Simon, you know, like street homelessness, using the shelter, living in a hostel and that sort of thing?

Doing this consistently gave the discussion a coherence that it might otherwise have lacked. Second, the character was used repeatedly as a means of re-focusing the discussion when it started to stray too far from the point, when one participant threatened to dominate by talking too persistently about his own situation, or when a participant had disclosed something very personal and might have been feeling uncomfortable. In each case, a member of the research team quickly shifted the focus of attention back to Simon, or from “I” to “he.” For example,

P-R: Sorry for interrupting. So how do you think Simon feels on that? What do you think might be going through his head?

Used in this way, the fictional character provided a safe refuge for both participants and researchers alike, and also encouraged the group to think in more generalizable terms, rather than simply recounting their individual experiences.

### Group 2

Group 2 was very different insofar as the two participants’ discomfort with the group setting and with each other was apparent from the outset. In the absence of a peer researcher, the character-building exercise was facilitated by a male researcher (M-R), who struggled to persuade them to choose a picture. Eventually they selected a young Black male, whom they named John. Both participants were very reticent, volunteering nothing and giving terse, monosyllabic responses when questioned. They seemed to have little interest in the activity until, when asked what John had been in prison for, one suggested that he was a hostage-taker. This finally produced some perfunctory laughter, as did a suggestion that his mode of transport was a BMX. These ideas later served as valuable tools for members of the research team, who were able to re-introduce them periodically to inject humor into the session and lighten the mood. However, despite repeated prompting, the character remained thin and unconvincing, lacking substance and a “presence” in the room. Unlike Group 1, whose participants identified their fictional character as one of them and empathized with him, the Group 2 participants distanced themselves from John, taking a superior and mocking stance from the outset. Instead of constructing him “in their own image,” they created a caricature and figure of fun. Given their choice of a Black male, this may have been indicative of racial prejudice and posed an additional dilemma for the research team in terms of how they dealt with it. While staying silent might have implied endorsement of the participants’ attitudes, challenging their character-creation in any way would have jeopardized the precarious sense of group cohesion that the team had struggled to establish and would also have reinforced their position of power.

The impression of distancing was reinforced by the participants’ response to the audio-recording. Unlike in Group 1, the recording did not evoke any fellow feeling. Instead, the participants demonstrated a harsh and aloof attitude, blaming John for his own misfortunes:

A2: I’ve no sympathy for what he’s done.

B2: Yeah, I know. Stay away from me, innit [laughs].

Fac: So you’ve got no sympathy for the street robberies?

A2: And hostage as well.

[Laughter]

Fac: Right. So you’re disliking him because of that. What about the stuff he was saying about feeling low and feeling down?

A2: Go get some antidepressants.

B2: Yeah, it’s his fault really.

With more prompting, they continued in this vein, asserting in detached and dismissive tones that he should “Just go see a doctor” (B2) and “See a psychiatrist” (A2). This recommendation in relation to the fictional character was in stark contrast to the way in which they both presented themselves, namely as highly self-reliant and help-avoidant. After some time, during which the facilitator tried in a number of different ways to draw them out, B2 offered a glimpse into his own history, emphasizing how he had managed to sort out his own problems and get himself to a position of relative stability, without help from anyone else:

B2: I basically been in that situation, innit. My dad died when I was thirteen. So I started drinking and stuff to take my mind off it and started gettin’ in trouble. And that’s it. I ended up going to prison and, but I haven’t had help for it, but I’ve sorted myself out. Yeah, I’ve just thought this is enough, I need to sort myself out, stay away from drink and that. And I’ve done it myself. But it’s took me years to do it.

After a while, A2 did the same, presenting himself as equally independent:

A2: Everyone really is thinking about themselves. So you’ve just got to learn to think of yourself all the time, innit. Take care of yourself. That’s what you’ve gotta do . . . I think it would make it worse, sitting there talking to someone about something that ’appened to me. I, I never talk about anything. My brother passed away a few years back. I didn’t do nothing about that. I just kept it to myself. That’s it.

So why might they be prescribing for John a set of behaviors (“seek help” and “talk to someone”) that neither of them professed to practice? One possible reason is that they were giving answers that were congruent with what they assumed the researchers’ views to be (a form of response bias), thus trying to please *us* while trying not to lose face *with each other* by allowing themselves to be seen as weak or emotionally needy. Wellings, Branigan, and Mitchell (2000) draw attention to the fact that focus group participants are often doing several different things simultaneously to achieve different purposes.

The social dynamics of this very small group were particularly interesting. On the surface, it appeared as though there was very little interaction between the two participants. Neither spoke except in response to a question from a member of the research team and then each one offered their separate opinion, without seeking to engage with the other or working toward a shared understanding or co-construction. They engaged in polite turn-taking, but in many ways the session resembled two individual interviews being conducted in the same room at the same time, each question eliciting a response from first one participant and then the other:

Fac: If you were feeling rubbish, who would you talk to?

A2: I, I never do. I just keep it in.

B2: I’d probably speak to my missus, innit.

Both participants routinely ended their sentences with “innit” (a contraction of “isn’t it?”), but this appeared to be a purely rhetorical device (a sort of verbal tic) rather than intended to elicit support or agreement from the other, as would normally be the case with such tag questions. Nor did it bring forth such a response. Despite spending over an hour in the room together and although there was some shared humor, the two young men established no obvious rapport with each other and there was little explicit agreement or disagreement; they appeared to remain for the most part in parallel universes. Thus, as a focus group, the object of which is to encourage individuals to interact *with one another* rather than with the researcher (Kitzinger, 1994), this session might have been judged a failure.

Nevertheless, close reading of the data revealed that there was no lack of interaction between them; they were just not interacting in the way that we expected them to. Instead of the conventional exchange of ideas, there was a great deal of what we took to be posturing going on between the two young participants. This began during the fictional character-building exercise, with the two lads seemingly competing with each other to come up with increasingly silly and outlandish attributes, which resulted in a wholly unconvincing character. It then continued with each of them striving to present himself as tough and resourceful, demonstrating that he spoke the right lingo and could lay claim to membership of a particular youth subculture.

While Group 1 participants had *talked* a great deal about men’s tendency to “put on a front,” there was little evidence of pretense or posturing being performed in that group. Its participants talked openly to one another about their vulnerabilities, acknowledging that they had all been down the same road and recognizing the peer researcher as having been there too. By contrast, participants in Group 2 seemed to be *doing* “putting on a front” in the focus group. Their studied detachment, their refusal to take the character-building task seriously, and the game of one-upmanship they were playing all seemed to be part of an exercise in “impression management” (Goffman, 1971), possibly designed to mask their lack of confidence and their discomfort with the focus group setting. That diffidence was nonetheless betrayed by the *manner* of their talk. They both kept their voices low and muffled throughout, with the result that the transcript from this group remained incomplete despite repeated listening.

In this group, it was not what the participants said but how they performed that was truly enlightening, providing a powerful demonstration of the way in which young men *do* non-engagement with services. Thus, far from being a failure, this strange small group encounter was of profound significance and generated insights that were fed directly into the ENGAGER intervention manual. Without the initial stimulus of the fictional character-building exercise, it is possible that this performance would not have taken off in the way that it did. As in Group 1, the fictional character/caricature also provided the research team with an invaluable tool for moving the session along, introducing new topics, breaking silences, and resolving potentially awkward situations.

### Group 3

Group 3’s fictional character also lacked depth, but for entirely different reasons. The participants picked an older White male and were willing to supply specific details on request (name, age, girlfriend, kids) but did not volunteer any further information about him or show any interest in the exercise. However, a prompt by the facilitator to consider what football team he supported triggered a light-hearted altercation that broke the ice and brought participants and researchers together in a discussion of the previous night’s football scores.

The participants then listened to the audio-recording but it was abundantly clear that this group of very recently released men had no time for the hypothetical character (Bob) and his problems, having far too many urgent issues and too much distress of their own to deal with. They evaded questions about Bob and, with no prompting, launched straight into a poignant discussion of the challenges they and people they knew had faced on release and were still facing, due to system failures, lack of co-ordination between agencies, and apparent indifference on the part of individual officers. Participant A3 initiated this, pouring out the story of his shambolic release day:

A3: But the day I got out, they were going to take me back the same night, ’cause they weren’t expecting me in [city]. Which was a nightmare, yeah?

Fac: So who was going to take you back?

A3: Erm, well, I turned up in [city], two o’clock to see probation . . . and they phoned up the place I was meant to be going to . . . And they were like, “Well we weren’t expecting him today.” So I’m out on tag . . . and they said, “Well I’m sorry, we’re going to have to, like, send you back to prison. You’re going to have to wait here basically until you get arrested.” So I sat in probation . . . and I was worried about my script [prescription for methadone, a heroin substitute] for the next day, ’cause in [prison] I was getting it at half past eight every morning . . . Then they come and said to me, “They can’t arrest you ’cause they ain’t got the paperwork for it. So they’re gonna have to give you a safe sleep for tonight.” So I was like, “Can’t I just go back with my parents? You know, back to [town]?” But they were like, “No,” I can’t do that. So I went to this place for a safe sleep. It was a shit-hole when I got there. You know, I’d nothing, no blankets, no nothing . . . There was drug paraphernalia there, it was horrible . . . It took me half a dozen bin liners to clear all the stuff from the floor. It was disgusting . . . And er, so I woke up the next morning not knowing what was going on . . .

The other participants were equally ready to share their troubles. The discussion lasted a full hour and there was barely any lull in the conversation, during which the participants remained resolutely focused on their own immediate concerns, including the inadequacy of their discharge grant to cover even the basics of survival, the lack of help with negotiating the benefits system, the impossibility of finding work in the week prior to Christmas, and the shame and heartache of being unable to buy presents for their children.^4^

So enmeshed were they in their current difficulties that they were completely unable to rise out of their own first-person perspective and see the world from Bob’s point of view, or shift from “I” to “he” and back again as participants in the first two groups were able to do. Thus, the fictional character played absolutely no part in this group discussion, but was tossed aside as an irrelevance. That this was a consequence of the participants’ current crisis and not indicative of any lack of compassion or fellow feeling was evident from the fact that they showed genuine concern for each other:

B3: I’ve, I’ve suffered really badly with anxiety since I come out this time, and . . .

D3: I think, I think you do though, when you come out of jail. Cor, my anxiety levels the first week I was out was through the roof. I was a nervous wreck I was. But once you get over that it gets a lot easier, you know what I mean?

B3: Yeah but I can’t, but I can’t, like doing this today. I’ve only, the only reason I’ve done this today is cos I’ve had to see my prolifics worker and . . . she said, “Do you want to do this thing this afternoon?” Otherwise I’d still be sat at home now thinking I don’t want to go out today.

D3: Yeah. But you did. You’re doing all right, aren’t yer?

B3: Yeah. Yeah, I suppose I am doing alright, yeah. But at the same time as that, I’d like to be doing better.

There was an altogether different quality of interaction between the men in this group. Perhaps recognizing their shared vulnerability, they were gently coaxing and offering encouragement to each other throughout, with no element of competition whatsoever. There was also evidence of co-construction, with participants building on each other’s accounts to create a vivid picture of what life was like both in prison and on the outside:

A3: I mean I met a guy in [prison], he was being released in a few days, and . . . he was going to go and commit another crime just to come back in, ’cause it’s cold in winter. Get three meals a day in there. It was just easier.

B3: Sometimes it’s nice to have routine. You know what I mean?

A3: Yeah, definitely, definitely.

B3: Get up, get unlocked, get a shower, get banged up again. Get unlocked, get your dinner, get banged up again . . . And when you come out they give you all this freedom, but if you don’t know what to do with that freedom what’s the point in having it? . . . I come out on Friday last week. And for the past, since that day, since I come out I’ve just wanted to go back in, to be honest. That’s all I want to do is go back to prison. It’s just easier.

Their raw distress and defencelessness—so plain to see and creating such an immediate bond between the men in this group—not only made them unable to relate to a fictional character but also rendered the technique redundant. So impossible was it to hide their pain from each other, and so great was their need to share it, that the men did not need the fictional character, either to prompt them to talk or to act as a shield behind which to hide; to facilitate engagement or to protect against over-exposure. Their recognition of their shared predicament did the work of enabling them to feel safe with each other. The facilitator and co-facilitator, who considered themselves to be reasonably emotionally hardened to the experiences of the target population, both admitted during a longer than usual debriefing that they had found this session utterly heart-breaking.

### Other Ethical and Methodological Factors

A number of other factors may have contributed to differences between the groups and are worthy of note in relation to the safe and effective conduct of these focus groups. As noted earlier, peer researchers were only available for Groups 1 and 3, and a different peer researcher (P-R) was used for each. The P-R in Group 1 had previous experience of facilitating group work in prison and came across as confident and relaxed with the participants, establishing an easy rapport with them from the outset. He took complete charge of the character-building exercise, encouraging them to develop a fully rounded character, and then intervened at regular intervals to ask questions. He also made good use of the fictional character, referring back frequently to Simon and his situation:

P-R1: So say for instance this. Say Simon comes out of prison. He comes to [city]. He doesn’t know anything. He doesn’t know anybody. He doesn’t know where to go. How would, what, what, what type of thing do you think that will impact on his mental, on his mental state?

By contrast, the P-R in Group 3, while he had been keen to help with the group to develop his skills and confidence, was noticeably less self-assured. He asked far fewer questions, thus possibly contributing to the side-lining of the fictional character. Instead, he repeatedly became caught up in the participants’ troubles-telling, corroborating their stories and sharing his own, and acting more as peer than as researcher. This highlights the complexity of the peer researchers’ role and the difficulty they faced in occupying the boundary between participants and researchers and maintaining dual identity. As others have noted, peer research is far from straightforward (Lushey & Munro, 2015).

The importance of the peer researcher was demonstrated in Group 2, in which a male member of the wider project team acted as proxy. This addressed the issue of gender but failed to address that of power, which was made more acute by the composition of this very small group (three researchers to two participants). His status as an academic researcher and his lack of lived experience of being subject to the criminal justice system was obvious from the questions he interposed during the discussion, which were clearly those of an outsider, such as: “Is money quite a big issue then?” It is impossible to know to what extent the tension in this group, the posturing, and the preposterous fictional character were due to the absence of a P-R, but it is likely that this contributed to the awkwardness of the encounter.

The same facilitator and co-facilitator conducted all three groups. For both, the biggest challenge was to come across as warm and friendly, while maintaining a safe and respectful distance, establishing enough authority to run the group and achieve its aim, and managing participants’ expectations, which included making it clear that the research team could not help them resolve their life difficulties. Although it was evident that she did not share a common background with the participants, the facilitator worked hard to reduce the sense of social difference. She was aided by a strong regional accent, an overall demeanor that would not be perceived as “posh,” a knowledge of football, and an innate sense of humor, all of which she utilized freely. Like the peer researchers, both of whom drew attention to their poor handwriting on the flip chart, the facilitator used self-deprecating talk to reduce the power differential between herself and the participants, repeatedly emphasizing her lack of technical mastery with regard to the recording equipment:

Fac: We’ve got [fictional character] pretending to speak on here now, to tell you summat different about him—if I can press the right button.

She also worked hard to strike the right balance between knowing and not knowing, demonstrating enough knowledge about the topic area to establish her credibility in the eyes of participants, while also acknowledging their expert status and insider knowledge. The former was achieved by sharing what she had learned from previous informants in this and other studies (e.g., “Some of the other guys I’ve talked to have said. . .”), the latter by naïve questioning and active listening techniques.

The presence of a co-facilitator is not apparent from the transcripts, as she played no part in the discussion, but this role was vital in terms of administering the groups, buying food, attending to the emotional welfare of participants, observing and making notes during the groups, and debriefing afterwards. Like the peer/male researchers, the co-facilitator was someone with whom participants could chat informally during the tea-break and after the session and may have been seen as more approachable than the facilitator, who was clearly in charge.

Although rarely reported in the literature, what happened “offstage” (that is, during the consent process, before the start of the session and during the break) was almost as important as the on-stage “work” of group discussion. These less formal times provided an opportunity not only to build rapport but also, through the provision of food and warm drinks and the act of eating together, to demonstrate care and respect for members of this normally marginalized group. At the same time, they contained many hazards, some residing in the smallest of details, such as inadvertently emphasizing social inequalities by providing brands of food and drink that the participants would have been unable to afford. Attention was drawn by the peer researchers to the fact that what we might see as a treat could easily backfire and reinforce the participants’ sense of inadequacy.

## Discussion

In an effort to help men with lived experience of being subject to the criminal justice system to participate in focus groups on the topic of mental health, while also managing specific challenges associated with this seldom-heard group, we used a novel technique involving the creation of a fictional character, supplemented by an audio-recorded vignette. We studied the role played by this technique in achieving our stated aims of *engaging* our participants without *exposing* them to undue risks.

In our first group, the fictional character technique functioned much as we had anticipated. Participants bonded as a group during the character-building exercise, and in the subsequent discussion were able to switch easily between first- and third-person discourse. This offered them the freedom either to own their experiences and mental health issues (using “I” talk) or to disown them by projecting them onto the hypothetical character (using “he” talk), thus avoiding direct disclosure and saving face among their peers if they wished to do so. This group of men, who had experienced homelessness but were now settled and who demonstrated considerable maturity, were both willing to talk openly about themselves and able to put themselves in the shoes of the fictional other.

Our second group raised a number of issues, chief among them the question of whether this was a focus group at all or rather a dyadic interview. Toner (2009) gives a powerful defense of very small focus groups (VSFGs), reporting how in groups of only two participants she succeeded in creating a climate of intimacy that resulted in strong affective bonds between participants and thick narrative data. Morgan and colleagues, by contrast, draw a distinction between dyadic and triadic interviews (two and three participants respectively) and focus groups (four or more participants), all of which are distinguished from individual interviews by the process of sharing, comparing, and extending of viewpoints that occurs through interaction between participants (Morgan, Ataie, Carder, & Hoffman, 2013). Given the nature of the interaction between participants in our second group, we may question whether it even qualified as a dyadic interview. To a large extent, the participants remained two individuals, each expressing his own “held truths” rather than working together to negotiate new ones and co-construct meaning through interaction (Belzile & Öberg, 2012). As for the fictional character technique, while it appeared to fail in this group as neither participant showed any readiness to engage with the figure of fun they had created, it did provide the research team with a point of reference to help them navigate this difficult social encounter, and it generated crucial learning about the way in which young men in the criminal justice system “perform” to service providers and to each other. This demonstrates well the point made by Wellings et al. (2000), namely that apparent defects in focus group process can be treated as data, rather than as disasters. We also strongly believe, like Toner (2009), that to have canceled the focus group because only two people showed up would have been to collude in silencing the participants and the seldom-heard group that they represented.

In our third group, the fictional character technique again appeared on the surface to fail insofar as the participants, very recently released from prison and overwhelmed by their own immediate difficulties, were completely unable to project these onto a hypothetical other. The exercise required participants to step outside themselves and imagine a situation from another’s point of view, and this proved to be impossible for Group 3 participants at this particular time. They were too caught up in the chaos of the moment. However, their inability to enter into the exercise was itself highly instructive, telling us more about the participants’ situation and the challenges that release from prison poses than if they had played along in a tokenistic way. On our part, *allowing* the technique to fail was therefore pivotal. Permitting this group to discard the fictional character because their own needs lay elsewhere played a vital part in keeping them engaged and enabling them to feel properly heard, while also generating important learning about what really mattered to them.

In the course of wrestling with these data, we have found ourselves challenging normative assumptions about what constitutes a “good” focus group. Conventional wisdom suggests that it should include at least four participants, who focus on the topics and tasks that are set and who all speak equally and clearly so that an accurate transcript can be produced. In two out of our three groups, we appeared to fail in one or more of these respects. Our findings show not only how unruly real-life data collection can be, but also that participants’ refusal or inability to focus on a task can be revealing in itself and that at times it may be necessary to abandon our own procedures and allow members of a normally silenced group the freedom to dictate what happens and to express themselves in their own ways. We believe that the pursuit of scientific rigor should never outweigh the rights of participants to feel truly accepted and valued.

## Conclusion

Using a novel technique involving the creation of a fictional character as a focus for discussion, we succeeded in eliciting valuable data from groups of men who are seldom given an opportunity to influence the development of services, while also doing our utmost to protect them from any discomfort or risk of exploitation. We are not aware of any adverse effects arising from the focus groups. We conclude that the technique would lend itself to being used in focus groups with other marginalized or seldom-heard populations. While we developed it in the course of working with men, the technique is not gender-specific and it would be interesting to know how it performed with a female population or with mixed groups. We therefore invite others to test it with different groups and report their findings.

## Appendix

### Text of Audio-Recording

*It was a few years ago that I began to feel different from all my friends. I felt that I couldn’t tell no-one anything ’cos all my mates would just laugh at me. My family knew something was up but I just kept saying I’m fine. But I wasn’t fine. I felt worthless, and that nobody or nothing could make me stop feeling like shit. Just getting up in the mornings was a struggle ’cos nothing seemed to interest me.*

*Once all my mates was getting a Mrs and starting work, all I wanted to do was hide away from people. I missed my 18th birthday. My friends and family said that I should have a party but all I wanted to do was sit in my room and watch telly.*

*People don’t understand what it’s like to be me. When you feel like you have nothing to live for. Depression makes me feel different from others, not normal. It’s like not having control of the things happening around you. I felt worthless and at times I hated myself. I had times when I felt very happy, followed by feeling very low, which can last up to days or weeks.*

*Now I’ve had help in dealing with my feelings and I can understand what’s happening and what to do when I feel this way.*

## Notes

1. Members of this group are more commonly referred to as “offenders” or “ex-offenders,” but this language is also problematic. Those who have served their sentence should not continue to be labeled in “offender” terms. Others may not have offended at all; they may have been on remand and subsequently acquitted.

2. The intervention was being developed for men only. In the United Kingdom, female prisoners are fewer in number, are housed in prisons further from their homes, and have very different resettlement needs. The ENGAGER research team plans to adapt the intervention for women in future, subject to trial results (Kirkpatrick et al., 2018).

3. In all cited passages, we indicate speakers using the following abbreviations: facilitator (Fac); co-facilitator (Co-fac); peer researcher (P-R); male researcher (M-R). Participants are indicated by an individual identifying letter and their focus group number: thus, C1 is Participant C in Focus Group 1.

4. For one participant, the high-street shopping voucher that we offered as a “thank you” represented his only hope of buying Christmas presents for his children.

## References

[bibr1-1049732318785359] Aldridge J. (2014). Working with vulnerable groups in social research: Dilemmas by default and design. Qualitative Research, 14, 112–130. doi:10.1177/1468794112455041

[bibr2-1049732318785359] Barbour R. (2007). Doing focus groups. Thousand Oaks, CA: Sage.

[bibr3-1049732318785359] Belzile J. Öberg G. (2012). Where to begin? Grappling with how to use participant interaction in focus group design. Qualitative Research, 12, 459–472. doi:10.1177/1468794111433089

[bibr4-1049732318785359] Britton S. (2012). Why priorities young adults? London: Barrow Cadbury Trust Retrieved from https://www.barrowcadbury.org.uk/wp-content/uploads/2012/09/T2A_PCC-briefing-Ver31.pdf

[bibr5-1049732318785359] Brooker C. Repper J. Beverley C. Ferriter M. Brewer N. (2002). Mental health services and prisoners: A review. Sheffield: School of Health and Related Research, University of Sheffield.

[bibr6-1049732318785359] Burns S. Schubotz D. (2009). Demonstrating the merits of the peer research process: A Northern Ireland case study. Field Methods, 21, 309–326. doi:10.1177/1525822X09333514

[bibr7-1049732318785359] Byng R. Quinn C. Sheaff R. Samele C. Duggan S. Harrison D.. . . Campbell J. (2012). COCOA: Care for Offenders, Continuity of Access. National Institute for Health Research Service Delivery and Organisation Programme.

[bibr8-1049732318785359] Cohen M. Garrett K. (1999). Breaking the rules: A group work perspective on focus group research. British Journal of Social Work, 29, 359–372. doi:10.1093/oxfordjournals.bjsw.a011462

[bibr9-1049732318785359] Dickson-Swift V. James E. Kippen S. Liamputtong P. (2007). Doing sensitive research: What challenges do qualitative researchers face? Qualitative Research, 7, 327–353. doi:10.1177/1468794107078515

[bibr10-1049732318785359] Goffman E. (1971). The presentation of self in everyday life. London: Penguin.

[bibr11-1049732318785359] Gott M. Seymour J. Bellamy G. Clark D. Ahmedzai S. (2004). Older people’s views about home as a place of care at the end of life. Palliative Medicine, 18, 460–467. doi:10.1191/0269216304pm0269216889oa 15332424

[bibr12-1049732318785359] Hodgins M. Millar M. Barry M. (2006). “. . . it’s all the same no matter how much fruit or vegetables or fresh air we get”: Traveller women’s perceptions of illness causation and health inequalities. Social Science & Medicine, 62, 1978–1990. doi:10.1016/j.socscimed.2005.1908.1052 16213642

[bibr13-1049732318785359] Holley J. Gillard S. (2018). Developing and using vignettes to explore the relationship between risk management practice and recovery-oriented care in mental health services. Qualitative Health Research, 28, 371–380. doi:10.1177/1049732317725284 28836488

[bibr14-1049732318785359] Howerton A. Byng R. Campbell J. Hess D. Owens C. Aitken P. (2007). Understanding help seeking behaviour among male offerenders: Qualitative interview study. British Medical Journal, 334, 303–306. doi:10.1136/bmj.39059.594444.AE 17223630 PMC1796718

[bibr15-1049732318785359] Jayne R. (2006). Service user engagement in prison mental health in-reach service development. Mental Health Review Journal, 11(2), 21–24. doi:10.1108/13619322200600016

[bibr16-1049732318785359] Jefferson G. (1981). The rejection of advice: Managing the problematic convergence of a “troubles-telling” and a “service encounter.” Journal of Pragmatics, 5, 399–422. doi:10.1016/0378-2166(1081)90026-90026

[bibr17-1049732318785359] Johnson J. Oliffe J. Kelly M. Galdas P. Ogrodniczuk J. (2012). Men’s discourses of help-seeking in the context of depression. Sociology of Health & Illness, 34, 345–361. doi:10.1111/j.1467-9566.2011.01372.x 21707661

[bibr18-1049732318785359] Kidd P. Parshall M. (2000). Getting the focus and the group: Enhancing analytical rigor in focus group research. Qualitative Health Research, 10, 293–308. doi:10.1177/104973200129118453 10947477

[bibr19-1049732318785359] Kirkpatrick T. Lennox C. Taylor R. Anderson R. Maguire M. Haddad M.. . . Byng R. (2018). Evaluation of a complex intervention (Engager) for prisoners with common mental health problems, near to and after release: Study protocol for a randomised controlled trial. BMJ Open, 8, Article e017931. doi:10.1136/bmjopen-2017-017931 PMC5879493 29463586

[bibr20-1049732318785359] Kitzinger J. (1994). The methodology of focus groups: The importance of interaction between research participants. Sociology of Health and Illness, 16, 103–121. doi:10.1111/1467-9566.ep11347023

[bibr21-1049732318785359] Lushey C. Munro E. (2015). Participatory peer research methodology: An effective method for obtaining young people’s perspectives on transitions from care to adulthood? Qualitative Social Work: Research and Practice, 14, 522–537. doi:10.1177/1473325014559282

[bibr22-1049732318785359] Macnaghten P. Myers G. (2011). Focus Groups. In Seale C. Gobo G. Gubrium J. Silverman D. (Eds.), Qualitative research practice (pp.66-80). London: Sage.

[bibr23-1049732318785359] Morgan D. Ataie J. Carder P. Hoffman K. (2013). Introducing dyadic interviews as a method for collecting qualitative data. Qualitative Health Research, 23, 1276–1284. doi:10.1177/1049732313501889 23925406

[bibr24-1049732318785359] Morrow E. Boaz A. Brearley S. Ross F. (2012). Handbook of service user involvement in nursing & healthcare research. Chichester, UK: Wiley-Blackwell.

[bibr25-1049732318785359] Onwuegbuzie A. Dickinson W. Leech N. Zoran A. (2009). A qualitative framework for collecting and analyzing data in focus group research. International Journal of Qualitative Methods, 8, 1–21. doi:10.1177/160940690900800301

[bibr26-1049732318785359] Pearson M. Brand S. Quinn C. Shaw J. Maguire M. Michie S.. . . Byng R. (2015). Using realist review to inform intervention development: Methodological illustration and conceptual platform for collaborative care in offender mental health. Implementation Science, 10, Article 134. doi:10.1186/s13012-015-0321-2 PMC4584430 26415961

[bibr27-1049732318785359] Peek L. Fothergill A. (2009). Using focus groups: Lessons from studying daycare centers, 9/11, and Hurricane Katrina. Qualitative Research, 9, 31–59. doi:10.1177/1468794108098029

[bibr28-1049732318785359] Pratt D. Piper M. Appleby L. Webb R. Shaw J. (2006). Suicide in recently released prisoners: A population-based cohort study. The Lancet, 368, 119–123. doi:10.1016/S0140-6736(1006)69002-69008 16829295

[bibr29-1049732318785359] Puchta C. Potter J. (2004). Focus group practice. London: Sage.

[bibr30-1049732318785359] Robson P. Sampson A. Dime N. Hernandez L. Litherland R. (2008). Seldom heard: Developing inclusive participation in social care. London: Social Care Institute for Excellence Retrieved from http://www.scie.org.uk/publications/positionpapers/pp10.pdf

[bibr31-1049732318785359] Ryan S. Hislop J. Ziebland S. (2017). Do we all agree what “good health care” looks like? Views from those who are “seldom heard” in health research, policy and service improvement. Health Expectations, 20, 878–885. doi:10.1111/hex.12528 28160350 PMC5600235

[bibr32-1049732318785359] Tolich M. (2009). The principle of caveat emptor: Confidentiality and informed consent as endemic ethical dilemmas in focus group research. Journal of Bioethical Inquiry, 6, 99–108. doi:10.1007/s11673-11008-191241673

[bibr33-1049732318785359] Toner J. (2009). Small is not too Small: Reflections Concerning the Validity of Very Small Focus Groups (VSFGs). Qualitative Social Work, 8(2), 179-192. doi:10.1177/1473325009103374

[bibr34-1049732318785359] Wellings K. Branigan P. Mitchell K. (2000). Discomfort, discord and discontinuity as data: Using focus groups to research sensitive topics. Culture, Health & Sexuality, 2, 255–267. doi:10.1080/136910500422241

